# Book Reviews

**Published:** 1907-07

**Authors:** 


### BOOK REVIEWS.

PROGRESSIVE MEDICINE. A quarterly digest of the advances, discoveries and improvements in the Medical and Surgical Sciences. Edited by Hobart Amory Hare, M.D., and H. R M. Landis, M.D. Lea Bros. & Co., Philadelphia.

The June number devotes its first 55 pages to Hernia, which is ably presented by William B. Coley, M.D. Surgery of the Abdomen exclusive of hernia is taken up by Edward Milton Foote, M.D., and 104 pages are required for this important subject.

The next 100 pages are by John Clark, M. D., on Gynecology and particularly interesting and instructive is the review of the recent work on cancer.

Diseases of the blood, diathetic and metabolic diseases and diseases of the spleen thyroid gland, and lymphatic sytsem are handled in a scholarly way by Alfred Stengel, M.D.

This volume concludes with the subject of Opththalmology by Edward Jackson, M.D.

---

HARE'S THERAPEUTICS. A Text-book of Practical Therapeutics, with Especial Reference to the Application of Remedial Measures to Disease and their Employment upon a Rational Basis. By Hobart Amory Hare, M.D., B.Sc., Professor of Therapeutics and Materia Medica in the Jefferson Medical College of Philadelphia, Physician to the Jefferson Hospital, etc. New (12th) edition, enlarged and thoroughly revised to accord with the eighth decennial revision of the U. S. Pharmacopœia. In one octavo volume of 939 pages, with 114 engravings and four colored plates. Cloth, $4.00 ,net; leather, $5.00, net half morocco, $5.50, net. Lea Brothers & Co., Philadelphia and New York, 1907.

---

SCHLEIF'S MATERIA MEDICA AND THERAPEUTICS. A Pocket Text-Book of Materia Medica, Therapeutics, Prescription Writing, Medical Latin and Medical Pharmacy. By William

Schleif, Ph.G., M.D., University of Pennsylvania, Philadelphia. New (3d) edition, 12 mo. 470 pages. Cloth, $2.50, net. Lea Brothers & Co., Philadelphia and New York, 1907.

The simultaneous appearance of two books on different phases of a large subject renders it advantageous to consider them together. "Schleif" is primarily a text book on the fundamentals, devoting most of its space to Materia Medica, Pharmacology, Prescription Writing, Medical Latin, etc. "Hare" is equally a book for students and also for practitioners. It devotes one-half of its space to remedial agents, including both drugs and non-medical measures as well as foods, and the remaining half to diseases and their treatment, with copious and precise directions. Both parts are alphabetically arranged and fully cross-referenced, so that all information on any point can be quickly found. The "Index of Diseases and Remedies," with its suggestive annotations, is additionally helpful in practice. Dr. Hare's faculty for perceiving the pith of a matter and presenting it clearly is reflected in all his works and is the basis of their great popularity. For instance, this is the twelfth edition of his Therapeutics in seventeen years, and of most of the editions several large printings have been required. Though it is the second edition since publication of the new Pharmacopœia, the author has not been content with a perfunctory revision, but he has, on the contrary, scrutinized every line and made changes and improvements wherever necessary to represent his very progressive subject.

Reverting for a moment to these two books for the purpose of considering them together from the student's standpoint, it may be said that they fit into each other very advantageously. Present-day curricula divide the subject very much on the lines here followed: a course in the pure Materia Medica and annexa in the first years, and then a course on the clinical applications, or Therapeutics proper. These two books contain more information that it is possible to put conveniently into a single volume. They divide it conformably to the best practice in teaching, and together they cover the whole subject.

